# Five questions on the cell-to-cell movement of *Orthotospoviruses*

**DOI:** 10.1016/j.bbadva.2024.100124

**Published:** 2024-10-16

**Authors:** Pratibha Singh, Rishi Raj, H.S. Savithri

**Affiliations:** a Department of Botany, Mahatma Gandhi Central University, Motihari, Bihar 845401, India; b Department of Biochemistry, Indian Institute of Science, Bangalore 560012, India

**Keywords:** Plant virus, Negative strand RNA virus, Movement protein, Plasmodesmata, Protein trafficking

## Abstract

Plant viruses employ Movement proteins (MP) for their cell to cell spread through plasmodesmata (PD). MP modifies the PD and increases its size exclusion limit (SEL). However, the mechanism by which MPs are targeted to the PD is still unresolved and there is a lack of consensus owing to limited studies on their biochemical and structural characters. The non structural protein m (NSm) functions as the MP in *Orthotospoviruses*. Tospoviral NSm associate with ER membrane. They also form tubules in protoplasts. Groundnut bud necrosis virus (GBNV), a tospovirus, infects several crop plants throughout India and is economically very important. GBNV NSm associates with the membrane strongly *via* the C-terminal coiled-coil domain, modifies the membrane and causes vesicle fusion *in vitro* and remodels the ER network into vesicles *in vivo*. These vesicles are in contrast to the tubules formed by other related tospovirus in cells lacking cell wall. In this review, five important questions on the cell-to-cell movement of tospoviruses have been addressed and based on the various reports, a plausible model on the cell-to-cell movement of Orthotospoviruses is presented.

## Highlights

• Tospoviruses are enveloped negative ssRNA viruses.
• The replication and movement of these viruses are distinct from positive-strand RNA viruses.
• NSm, the movement proteins of tospviruses associate with ER membrane and alter the structure of ER network.
• Unlike other positive-strand RNA viruses movement proteins, NSm has a conserved C-terminal coiled-coil domain involved in membrane association, and vesicle formation and is important for a cell-to-cell movement.
• NSm also interacts with host factors that alter the ER network. However, the role of these vesicles in the viral life cycle is unknown.

## Introduction

Viruses enter a healthy plant either upon mechanical injury or when an insect vector harbouring the virus feeds on it. But successful infection of a plant depends on the ability of the virus to move from the initially infected cell to the neighbouring cells. Unlike animal cells, plant cells have a robust cell wall, which viruses cannot easily penetrate. The cell wall restricts the cross-connection between neighbouring cells, and the only specific cytoplasmic channel is the PD. The ability of plant viruses to cross the cellulosic cell wall to propagate infection throughout a plant has been a long-standing puzzle in plant cell biology and virology. MPs are non-structural proteins encoded by plant viruses that enable their movement from one infected cell to neighbouring cells.

The first insight into the mechanism by which viruses exploit PD for their movement came from studies on the MP encoded by tobacco mosaic virus (TMV; the 30-kDa protein) [1]. The TMV MP, when expressed in transgenic plants or when introduced by microinjection into leaf mesophyll cells, could increase the SEL of PD, and it appeared that cell-to-cell spread of infection could occur even in the absence of intact virion [2]. Subsequently, there were several similar investigations on other plant viruses and it soon became clear that MPs are a general feature of all the plant viruses and that they form complex with viral genomes which move from one cell to another via the PD [3].

The genus Orthotospoviruses (also called tospoviruses) of *Bunyavirale* order are the negative sense RNA plant viruses. They have three single stranded RNA genomes- L,M and S that encode for structural proteins such as RNA dependent RNA polymerase (RdRp), glycoprotein Gn and Gc and Nucleocapsid protein (N). The M and S RNA of tospoviruses have an ambisense coding strategy and encode non-structural protein NSm and NSs through complementary RNA strand that act as MP and suppressor of post transcriptional gene silencing respectively. Groundnut bud necrosis (GBNV) is the most prevalent and economically important tospovirus in India. It infects several Solanaceous and leguminous crops throughout the country [4]. Until recently, not much was known about GBNV NSm.

### History of NSm and other MPs

In tomato spotted wilt virus (TSWV), the type member of tospoviruses, NSm, was initially identified as a putative MP based on the observation that it associated with nucleocapsid protein (N) and was in close association with PD [5]. A year later, it was reported that TSWV NSm forms tubular structures in insect cells and protoplasts [6]. The induction of tubular structure in protoplasts by various MPs was reported as early as 1991 [7]. Further, the tubule carrying strawberry latent ringspot virus like spherical particles was also observed near PD in infected leaf section under electron microscope leading to the proposal that the tubular structures induced by MP were necessary for the cell to cell movement of the virus [8].However, the MPs from genus tobamovirus, tombusvirus, and hordeiviruses did not form tubular structure. Thus, the MPs were classified in to tubule forming and nontubule forming groups [9]. The MPs of different viruses lack similarity in their sequence but complement movement functions in MP deficient unrelated viruses suggesting that they have similar function but the mode of action could be distinct that may contribute to host specificity. TSWV NSm was able to complement cell-to-cell movement of a movement-defective TMV in plants [10]. This was the first functional demonstration that NSm is the TSWV MP [10]. In this review, based on the available literature on tospoviral NSm, five important questions concerning the mechanism of viral movement is presented.

Question 1 **Does NSm form tubules or vesicles?**

There have been many attempts to purify and understand the biochemical characteristics of the MPs from various viruses. But all MPs form an insoluble aggregate when expressed heterologously in *E.coli,* and their *in vitro* characterization have been performed with refolded proteins [11]. For example, TMV MP P30 can be purified to near homogeneity in the presence of urea and high salt concentration [12]. Similarly, cucumber mosaic virus (CMV) MP was purified in 8M urea and 2% triton X100 [13]. The refolded proteins do not always acquire the native structure. The lack of information on the structure of any MP makes the analysis even more difficult. Hence, the reports on the biochemical properties of MPs are limited and still need validation. For TSWV NSm also, *in vitro* studies like RNA binding and NSm-N protein interaction was accomplished by using refolded protein purified using 8 M urea [14].

GBNV NSm is the first MP to be expressed heterologously in *E.coli* and purified without addition of any denaturing agent (called as natively purified protein). The natively purified NSm is a soluble aggregate that resembles a vesicle like structure when observed under Electron microscope and are composed of bacterial membrane [15]. It also associate with artificial liposomes and are involved in fusion of artificial membranes *in vitro* (Fig. 2) suggesting that NSm has the ability to reshape membranes.

Likewise, *in planta*, many tospoviral NSm are strongly membrane-associated proteins. leading to the proposal that membrane association is a general feature of MPs of tospoviruses. These NSm more specifically associate and colocalize with ER network [[16], [17], [18], [19]]. However, GBNV NSm not only colocalizes with ER network but also remodels it into vesicles that agglomerate in the vicinity of the cell membrane (Fig. 2). Similar vesicles can also be observed in the leaf section expressing TSWV NSm. But, complete transformation of ER network in to vesicle does not take place and the structure has been proposed as reminiscent of ER network. (Fig. 3) [20,21].

Not only tospoviruses, but MPs of numerous positive strand RNA viruses like carnation mottle virus (CarMV), beet yellows virus (BYV) and melon necrotic spot virus (MNSV) have also been shown to be integral membrane proteins in planta [[22], [23], [24]].Vesicles have also been observed in *N. benthamiana* leaves transiently expressing MPs of many positive strand RNA viruses like potato virus X (PVX), turnip vein clearing virus (TVCV), potato leaf roll virus (PLRV) and hibiscus green spot virus (HGSV) [[25], [26], [27], [28]].

**Fig. 1:**
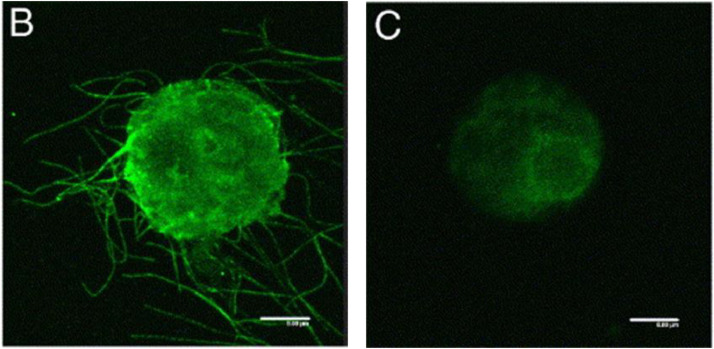
TSWV NSm forms distinct tubule in protoplast. Expression of NSm in tobacco protoplasts (B), Mock inoculated with water (C). Adapted with permission from Lewandowski and Adkins, 2005.

The presence of vesicle like structure in *E. coli* purified GBNV NSm and *in planta-expressed* GBNV NSm is in contrast to the observations on TWSV NSm that it forms tubules in cells devoid of cell wall like *tobacco* protoplast and insect cells [6] (Fig. 1). Such tubule formation is a general feature of MPs of some DNA and RNA viruses classified as tubule forming viruses Ex: caulimoviruses, umbraviruses, nepoviruses, tospoviruses and bromoviruses [29].

However, NSm has not been observed to form tubule at or near PD [16,26]. Further, tubule forming MPs can complement the cell to cell movement in unrelated viruses deficient in nontubule MPs. For example, MPs of nepoviruses, hordeiviruses, tobamoviruses and other bromeviruses can reestablish the Cell-to-cell movement of MP-deficient alfalfa mosaic virus (AMV) (bromovirus), demonstrating that the AMV viral genome can be transported from cell-to-cell by both tubule-forming and non-tubule-forming MPs [30]. Moreover, there are limited reports on attempts to understand the existence of such tubules at PD. Also, there is no clear observation and biochemical characterization of tubules loaded with virions have not been observed clearly and, asserting the lack of link between tubule and viral movement [10,31].

From the discussion above, it can be concluded that the NSm strongly associate with ER membrane and colocalize with ER network. The *in vitro* and *in vivo* reports on GBNV NSm membrane association provide mounting evidence on the ability of NSm to remodel ER network into vesicles. Although the tubule forming ability of MPs can be a general feature but the role of the tubule in cell-to-cell movement is not clearly understood. The tubules are only formed in the protoplasts and insect cell lines devoid of Cell wall and PD.

Question 2 **What domains of NSm are involved in membrane association and vesicle formation?**

**Fig. 2:**
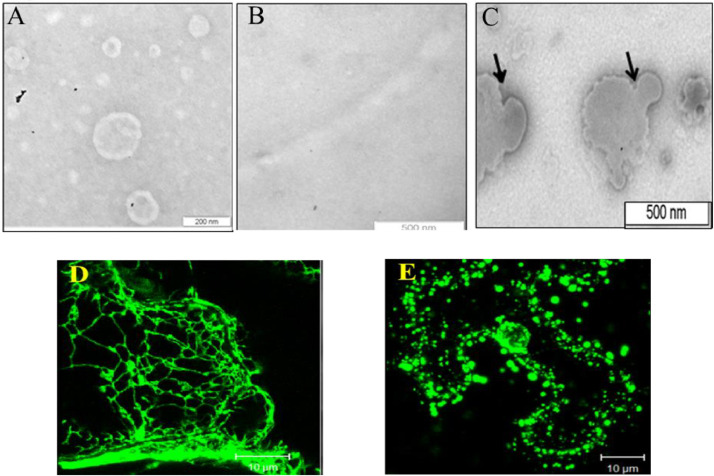
GBNV NSm has membrane remodelling ability *in vitro* and *in vivo*. Transmission electron Micrograph showing the interaction of refolded NSm with liposomes to form larger size of liposomes and the site of liposome fusion (shown by arrow) can be observed (A) Liposome alone, (B) Refolded NSm alone (C) Liposomes incubated with refolded NSm. (D) and (E) confocal images of N. benthamiana leaves expressing ERGFP (D) and ERGFP along with NSm. (Adapted from Singh et al., 2014 and Singh and Savithri, 2015).

The domains of MPs involved in membrane association are diverse. Several MPs have been reported to have one or two transmembrane domain (TMD) or hydrophobic regions involved in membrane association as summarised in Table 1. However, the *in silico* analysis of GBNV NSm suggests that it does not have any transmembrane domain. The membrane floatation assay of deletion mutants of NSm suggest that the C-terminal region is indispensable for membrane association [15]. Moreover, the localization of NSm to PD and remodelling of ER network is also terminated upon deletion of C-terminal domain [18] (Fig. 4). *In silico* analysis of NSm shows that it has a well conserved coiled coil domain at C-terminal region (Fig. 4**B**) across the species of tospoviruses and the C-terminal coiled coil domain alone also forms vesicles (Fig. 4**D**) in *N. benthamiana* leaves confirming that Nsm associates with ER-membrane through C-terminal domain and remodels it to vesicles (Fig. 2). This is the first report of any MP using coiled coil region for membrane association and remodelling [18]. At the same time, TSWV has been shown to associate with ER membrane using two hydrophobic regions HR1 and HR2 [17]. However, these regions are not conserved across tospoviruses, and mutation in the HR region does not bring NSm to the aqueous phase completely, suggesting that these regions could be partially anchoring the NSm to the membrane [17]. In another report about TSWV, The c-terminal coiled-coil region has been shown to be indispensable for cell-to-cell movement and tubular structure formation, suggesting a significant role of the coiled-coil region in the viral life cycle. The central domain is responsible for symptom development [10,32,33]. The above discussion clearly suggests that the conserved coiled-coil domain is directly involved in membrane association, as the coiled-coil region alone can also form vesicles.

**Table 1:** list of MPs from various viruses and their membrane associating regions.

**Movement protein**	**Domains involved in membrane association**	**Reference**
TuMV 6K_2_ (6 KDa protein)	One transmembrane domain	[43]
CWMV MP (37K protein)	Two transmembrane domains	[44]
TGB3 of PMTV	One transmembrane domain	[45]
MP of Melon necrotic spot virus (MNSV)	One transmembrane domain	[23]
TMV MP	Hydrophobic region	[46]
Closterovirus6-kDa MP (p6)	One transmembrane domain	[47]
Potato virus X (PVX) TGB2	two putative transmembrane domains	[48]
NucleorhabdovirusMPs	Associate with membrane by using hydrophobic region	[49]
GBNV NSm	C-terminal coiled coil domain	[15].
p29 and P24 citrus leprosis virus C (CiLV-C)	coiled coil domain	[50].
CaMV MP	C-terminal coiled coil domain required for PD localization	[51]

**Fig. 3:**
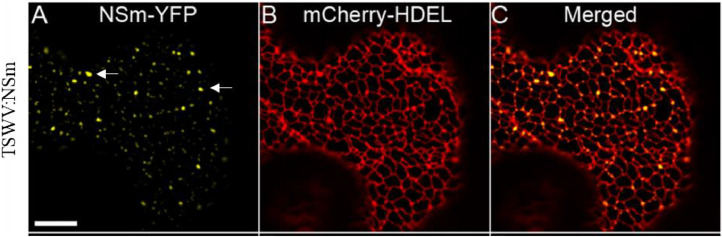
TSWV NSm localize to ER membrane at well defined places forming a structure similar to vesicle (A) Expression of NSm-YFP, white arrow indicates the structure similar to vesicle, (B) Expression of ER Marker mCherry-HDEL. (C) Overlay image (A) and (B) (© 2016 Feng et al. ) (Adapted from Feng et al, 2016).

**Fig. 4:**
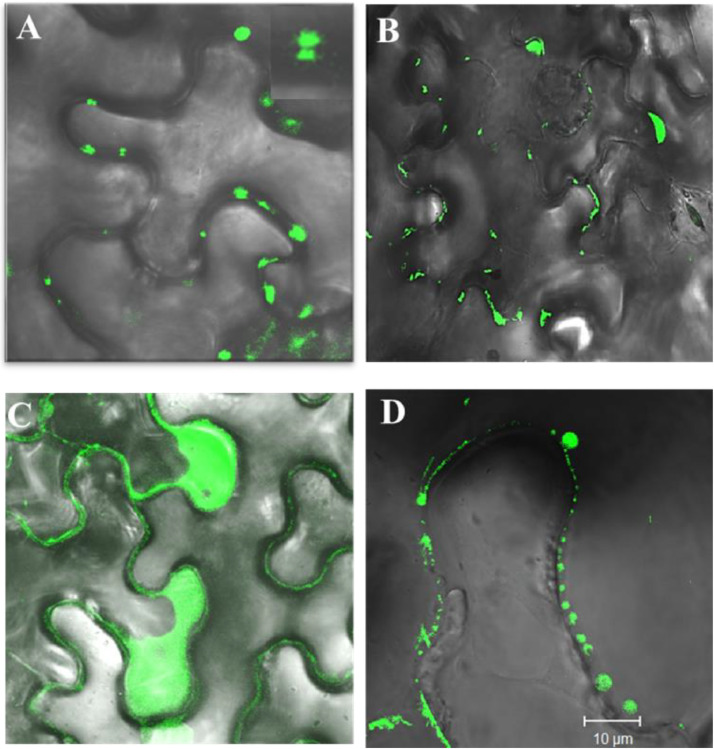
C-terminal coiled coil domain is responsible for ER localization of NSm.Subcellular localization of GBNV–NSm and its deletion mutants in N. benthamiana(Adapted from (Singh and Savithri, 2015) (A): GFP–NSm (B) GFP–NSm NΔ50 localizes to PD. The deletion of C-terminus region abrogates the localization of NSm to PD as shown (C) localization of GFP-NSm CΔ40. The C-terminal 79 amino acid possessing coiled coil domain form vesicle like structure near cell membrane as shown in(D) GFP-Nsm C79 aa.

**Fig. 5:**
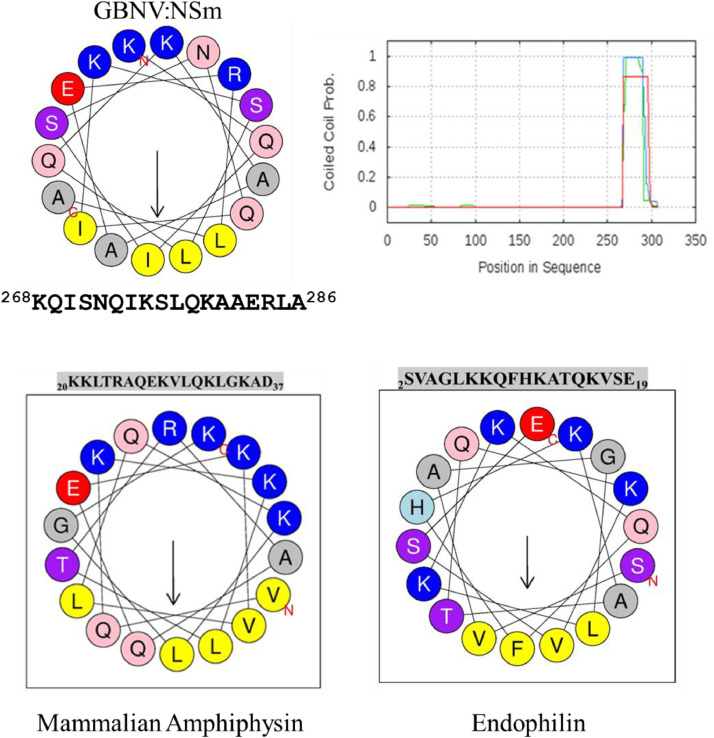
GBNV NSm has Amphipathic helix. Helical wheel projection of the α-helix from GBNV NSm protein in the C-terminal region, N-terminus region of Amphiphysin and Endophilin and coiled coil domain of NSm. Amphipathic helix was prepared by server Heliquest (https://heliquest.ipmc.cnrs.fr/). Red, acidic amino acid; blue, basic amino acid; yellow, aliphatic amino acid; purple and pink, polar amino acid prepared (A) C-terminus of NSm (C) and (D) Amphipathic helix of amhiphysin and endophilin by (B) coiled coil domain present at C-terminal region (Image adapted from Singh et al., 2014). Coiled coil domans are involved in membrane remodelling.

Coiled-coil domains harbour amphipathic α-helices with heptad repeats [34]. An amphipathic α-helix has a sequence of amino-acid residues that produces distinct hydrophilic and hydrophobic face. Such helices may associate with membrane via hydrophobic region and remodel them to vesicles by membrane fusion [[34], [35], [36]]. NSm also has amphipathic helix at C-terminal coiled coil region (Fig. 5)

The classical example of coiled coil domain is HA2 domain of hemagglutinin protein of influenza viruses [37]. Such coiled coil peptides involved in membrane fusion also exist in many other animal viruses like Moloney murine leukemia virus, Human immunodeficiency virus, Ebola viruses, and paramyxoviruses [37]. A related coiled coil structure is also present in v- and t-SNARE complex involved in synaptic fusion. In some proteins coiled coil regions having amphipathic helix are the component of BAR domain, a banana shaped tertiary structure that binds to membrane via their concave face. It is capable of sensing membrane curvature by binding preferentially to curved membranes [38] as observed in the case of Endophillin, Arfaptin2, amphiphysin a protein involved in promoting endocytosis in synaptic vesicle [38,39]. Interestingly,the BAR domain containing protein has also been shown to tubulate the membrane *in vitro* [38]. A comparative figure of amphipathic helix of Endophillin amphiphysin and NSm [[40], [41], [42]], is presented in Fig. 5.

In plant viruses, the p29 and P24 protein of citrus leprosis virus C (CiLV-C) also harbour coiled coil domain and have been suggested to be involved in their localization to PD and ER network remodelling to vesicles [50]. The MP of prunus necrotic ringspot virus (PNRSV), has an amphipathic helix that is responsible for membrane attachment [52].The role of C-terminal coiled coil structure in viral movement is also implicated in another tubule forming virus cauliflower mosaic virus (CaMV). CaMV MP C-terminal coiled coil domain is well conserved among all the caulimoviruses and is required for PD localization [51]. Brome mosaic virus (BMV) 1a protein (involved in replication) associates with ER membrane and remodels it into vesicles directly through C-terminal amphipathic helix [53]. Therefore, it is likely that coiled coil domains, in general, promote membrane localization and subsequent formation of tubules or vesicles. The coiled coil domain of GBNV NSm is clearly involved in ER remodelling and vesicle formation.

Question 3 **What is the role of endomembrane and cytoskeleton in movement?**

The Endomembrane system of a plant cell is made up of the endoplasmic reticulum (ER), Golgi apparatus, along with the trans Golgi network (TGN), various endosomes, and the vacuole [54].

**Fig. 6:**
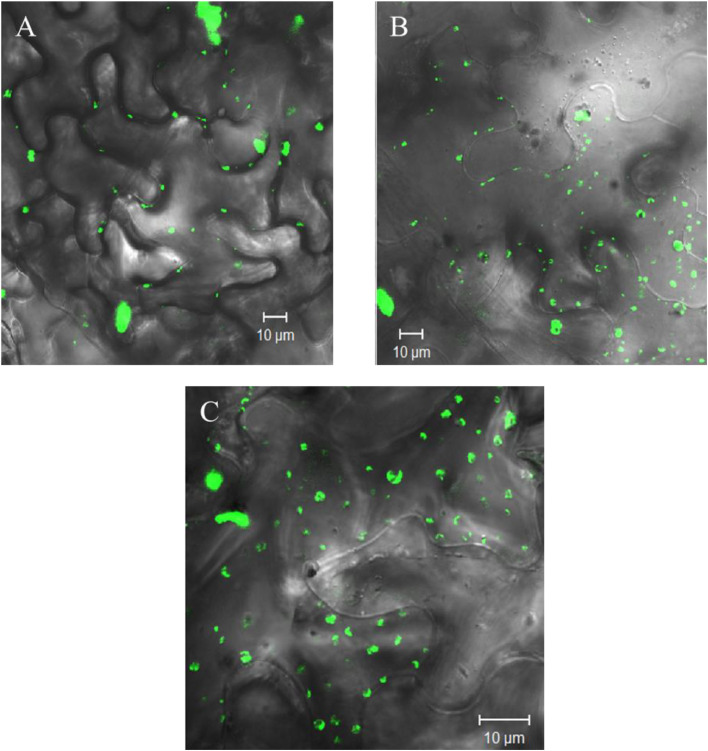
Tospoviruses use ER membrane system to transport NSm to PD. (Adapted from Singh and Savithri, 2015). Localization of GFP–NSm at PD (A), The localization of GFP NSm when the N. benthamiana leaves infiltrated with GFP:NSm construct are treated with BFA 10μg/ml (B) and 25μg/ml(C).

The GBNV NSm derived ER vesicles agglomerate in the vicinity of plasmodesmata. The localization of these vesicles to plasmodesmata is abolished in the presence of varied concentration of Brefeldin A (BFA) [18]. Moreover, the vesicles are deformed and aberrant in shape as shown in Fig. 6. BFA is a fungal toxin used to block secretory pathways in plants and animals. It binds to Guanine nucleotide Exchange Factor (GEF) and renders it inactive. Hence, the vesicle formation does not take place and the accumulation of SNARE protein in Golgi causes aberrant fusion of Golgi and ER network leading to formation of islands like structure. However, based on the BFA treatment result, it cannot be concluded that GBNV NSm vesicles enter Golgi network [[55], [56], [57]]. TSWV NSm do not co-localize with Golgi bodies, nucleus or chloroplast marker suggesting that similar to GBNV, it also utilizes ER-membrane transport system for its targeting to PD [21].

The ER is the largest membranous structure in the cell and unlike animal cells ER in plant cell is aligned with microfilament [58]. The ER undergoes continuous actin-based remodelling by tubule formation, elongation, sliding, and fusion, as well as continuous streaming [58].

Therefore, in order to understand the role of microfilament in NSm targeting, Latrunculin B, a toxin that binds actin monomers and block its polymerization [59] was used to understand the role of actin in targeting of NSm to PD. The localization of GBNV NSm derived vesicles were also blocked upon treatment with varied concentration of LAT B (unpublished observations). As ER is mainly associated with microfilaments in plant cells, it further strengthens the observation that microfilament may be involved in escorting ER derived \vesicles to PD [58].

In many positive strand plant viruses these ER derived vesicles or inclusion bodies, called Viral Replication Complex (VRC) or X-bodies are involved in genome replication and transcription. These vesicles colocalize with genomic RNA, RdRp (RNA dependent RNA polymerase) and capsid proteins. Such structures form a platform for sequestration of host proteins for efficient viral replication and protection from host defences [60]. Most often they use actin filament and myosin motor to move to PD [[61], [62], [63], [64]] suggesting that the replication and movement are linked events [27,60,[65], [66], [67], [68], [69], [70]].

The remodelling of ER network to vesicle is also prevalent in animal virus infection. In animal cells also, the vesicles are the site of virus replication. Such VRCs are mostly formed in positive sense RNA virus infection where the replication takes place in the cytosol. Likewise, although the ER derived vesicles are generated by many plant viruses, the presence of VRC in such vesicles are reported only for positive strand RNA viruses.

The members of *bunyavirales* to which GBNV belongs replicate in cytosol. However, so far, it has not been observed that replicating RNA organises itself in membrane bound VRC. Indeed, there are limited reports on the mechanism of replication of cytosolic negative strand RNA viruses. Recently, one of the bunyavirus SFTSV (severe fever with thrombocytopenia syndrome virus) was demonstrated to hijack autophagosome for its replication and viral emanation [71]. However, there are no reports on the mechanism and site of replication of tospoviruses.

The role of NSm remodelled vesicle in GBNV life cycle and its movement is not yet understood. The ER derived vesicles are also involved in secondary PD biogenesis [72]. Therefore, it is worth investigating whether vesicles are involved in PD biogenesis or whether they are the site of viral replication.

Question 4 **How does the interaction of NSm with other viral proteins/host factors modulate viral movement?**

The replication and movement of viruses depend highly on host protein interactions. MPs from various viruses interact with several host proteins associated with ER membrane, PD and cell wall. For example, TMV, turnip vein clearing virus (TVCV) and CaMV MPs interact with cell-wall-associated enzyme pectin methylesterase (PME) which helps in the movement of viral genome from one cell to other cell [73,74].

The TSWV NSm interacts with DnaJ homologue from tobacco (*Nicotiana tabacum*), Arabidopsis and tomato. Classical DnaJ proteins also called cochaperones are involved in regulation of HSP70, maintenance of protein homeostasis and transport across membranes [14]. DnaJ and its homologues localize to ER, cytosol, mitochondria and plasma membrane suggesting their versatile role in cell function. Further, the level of this protein increases in TSWV infection and cold stress indicating that the protein responds to biotic and abiotic stress. Another co-chaperone NbSGT1 from *N. benthamiana* also interacts with TSWV NSm and helps in viral spread [75]. Further, many other plant viral proteins like CP of potato virus Y (PVY) and potato virus X (PVX), CP of soybean mosaic virus (SMV), MP of rice stripe virus (RSV), MP of TMV have been reported to interact with DnaJ like proteins [[76], [77], [78]]. In these entire range of viruses, virus accumulation is positively regulated by the DnaJ and its homologues establishing that DnaJ could be a common host factor that facilitates virus accumulation. However, the region of NSm that interacts with DnaJ is unknown.

TSWV NSm also interacts with a protein called At-4/1 from *Arabidopsis thaliana*. The homologues of this protein are present in all plants. It shares similarity with myosin, kinesin andinteraptin, a protein linked with intracellular membrane compartments [79]. It is an alpha helical protein with two-third of it having coiled coil regions. The At-4/1 and its ortholog Nt-4/1 localizes to PD. Similar to NSm, At4/1 also remodel ER network into vesicles. Based on its ability to colocalize with MP near PD it is suggested that it may be involved in cell-to-cell communication [80].

Nt-4/1 forms aggregate similar to vesicle like structure in cytoplasm. The electron micrograph of *E.coli* expressed Nt-4/1 indicate that it forms large multimeric filamentous structure [81]. The protein also has five coiled coil domains of which coiled coil V present at C-terminus is conserved [82]. Coiled coil V is essential for the formation of filamentous structure [81]. Expression of Nt4/1 also induced the formation of vesicle like structure in N. benthamiana leaves. However, for this author has suggested that the NT4/1 is not membrane bound but associated with endosomes [81]. It accumulates in the veins at specific stage and positively regulate the multiplcation of virus [81]. Based on these reports it can be speculated that the tubular or filamentous structure of NSm observed in tobacco protoplast is due to the interaction of NSm and Nt-4/1. Further, as it also remodel ER network in to vesicles, there is a strong relation between NSm and Nt4/1 supporting the ability of tospoviral NSm to remodel ER network. As Expression of Nt-4/1 positively regulates the viral accumulation, it can further be speculated that NSm-Nt-4/1 interaction is vital for the intercellular movement of viruses. However, the mechanism of NSm-Nt-4/1 or At-4/1 interaction and the signals required to upregulate the accumulation virus is not understood.

Recently, the central region of TSWV NSm has been shown to interact with Resistance gene product Sw-5b involved in hypersensitive response [83]. The overexpression of SW-5b in *N. benthamiana* provide resistance against TSWV. Hence, Sw5b is involved in reducing the viral spread.

Besides MP- host factorsinteraction, MPs oligomerize and also collaborate with other viral proteins for cell-to-cell movement. NSm shows homotypic interaction and oligomerizes to form large multimeric complexes in INSV and in Iris yellow spot virus (IYSV) [20,84]. In IYSV NSm-NSm oligomerization,the interacting domains are from amino acid 1–160 [83]. NSm also interacts with nucleocapsid protein (N) through its N-terminal region [18,31,32]. The TSWV N Protein also shows homotypic interaction and forms an asymmetric trimeric ring. To form the trimeric ring, the N and C arms of the N protein interact with the adjacent two N proteins [85]. N Protein from TSWV and GBNV associates with RNA non-specifically [[86], [87], [88]]. Additionally, it has been reported that the negative strand RNA viruses move from one cell to other cell in the form of nucleocapsid RNA and RdRP complex further supporting the significance of NSm-N interaction [49,89]. The N protein also interacts with Gn (Glycoprotein N) and Gc (Glycoprotein C) in planta [90].

However, there is no report of interaction between NSs and NSm. NSs acts as a viral defence protein and suppresses PTGS induced by host. It binds to siRNA and has phosphatase, ATPase and helicase activity. It also enhances translation independent of helicase activity [[91], [92], [93]]. Being a multifunctional protein, NSs is also studied for its role in replication and movement besides being PTGS suppressor. TSWV NSs enhances baculovirus replication in insect cell lines, suggesting its possible role in replication [94]. Additionally, TSWV NSs supports infection and systemic movement of turnip mosaic virus, a potyvirus [95]. NSs has been reported to interact with N Protein and also shows homotypic interaction. NSs, Gc and Gn accumulate in nucleus as well as cell periphery [20,96].

To summarise, NSm interacts and colocalizes with two host factors Dna J and At-4/1. These two host factors positively regulate the virus accumulation indicating that they are involved in increasing the susceptibility of plant to viral infection. TSWV NSm interacts with Sw-5b, a protein involved in defence against pathogens. It also associates the trimeric N protein-RNA complex. These supercomplexesmay be targeted to PD by concerted effect of host protein and NSm via its C-terminal coiled coil domain.

Question 5 **Is phosphorylation/ dephosphorylation by host-encoded kinases a common mode of regulation of the viral life cycle?**

Post-translational modifications reshape the protein structure and function by adding a functional group or metal ions covalently. Such modifications regulate the stability, activity and subcellular localization of modified proteins. One such common modification, phosphorylation, switches the structure and activity of protein substrates by adding a charged and hydrophilic phosphoryl group [97].

MPs from TMV, PLRV, tomato mosaic virus (ToMV), potato virus X (PVX), also get phosphorylated by host kinases [[98], [99], [100], [101], [102]]. In addition, CP of positive strand RNA viruses also undergoes phosphorylation, as shown in the case of BMV, beet black scorch virus (BBSV), CaMV, potato virus A (PVA), plum pox virus (PPV), bamboo mosaic virus (BaMV), PVX [[103], [104], [105], [106], [107], [108], [109]].

At the biochemical level, phosphorylation reduces RNA binding. The viral proteins upon phosphorylation gain negative charge which perhaps result in dissociation of RNA-MP complex in the neighbouring cell. SatBaMV (satellite Bamboo Mosaic Virus) encoded MP P20 phosphomimic mutant failed to oligomerize and bind to RNA [110]. Further, PVX encapsidated gRNA cannot be translated until the CP interacting with the gRNA is phosphorylated by host kinase and unpackaging of RNA takes place [108]. Similarly, the phosphorylation of PVA and BaMV CP, at the RNA binding domain reduces their RNA binding affinity. With these reports, it can be concluded that phosphorylation by kinases reduces the affinity of CP towards RNA, leading to the release of gRNA to cytosol for further translation and replication.

The phosphorylation of viral proteins also alters their localization. The localization of phosphorylated MPs of ToMV, TMV, and PLRV is modified and infection is reduced suggesting that phosphorylation of MP affects viral spread [62,111,112].

GBNV NSm has maximum number of serines compared to all other amino acids (37 in number, 12% of total amino acids). *In silico* analysis also indicates 32 probable phosphorylation sites. However, till now the phosphorylation of NSm has not been reported. The role of phosphorylation of MPs in viral life cycle is not clear.

However, NP from GBNV gets phosphorylated by CKII and probably by other host kinases also [87]. The role of phosphorylation of NP in GBNV life cycle has not been elucidated. Based on the effect of phosphorylation on other capsid proteins it can postulated that Phosphorylation of NP might be involved in regulation of RNA binding.

## Conclusion

In spite of the observation that TSWV NSm induces tubules in protoplast and insect cell lines, the other orthotospoviruses NSm induced tubules have not yet been observed at plasmodesmata. The mechanism of tubule formation and their role in viral movement needs further studies. However, tospoviral NSm associates with ER membrane and GBNV NSm clearly remodels it to vesicles via the C-terminal coiled coil domain. Such use of coiled coil domain by MP is novel and points to the fact that MPs from various viruses use different strategies to perform similar cell to cell movement. Nonetheless, the cargo of vesicles in TSWV does not associate with Golgi network and is directly transported to PD by actin mediated pathway. In various positive ssRNA viruses such vesicles are the site of replication. However, in negative strand RNA viruses there is no such report. Vesicles have also been shown to be responsible for the formation of secondary plasmodesmata. Therefore, the role of vesicles in the replication and movement of viruses needs further investigation.

For successful infection plant virus interact with many host proteins. TSWV NSm interact with dnaJ homologues and At4/1 proteins that helps in accumulation of virus. Further, NSm also interact with host Resistance immune receptor factor SW-5b.It contribute to resistance against American strain of TSWV in tomato [113]. However,SW-5b resistance factor does not confer resistance against Asian tospoviruses like GBNV. Therefore, exploration of host factors that restrict the movement and replication of GBNV is a future prospect worth investigating.

MPs from various viruses undergo phosphorylation and modulate viral movement. The exploration of phosphorylation of NSm and its impact on viral life cycle can also give insight into viral movement regulation.

**Fig. 7:**
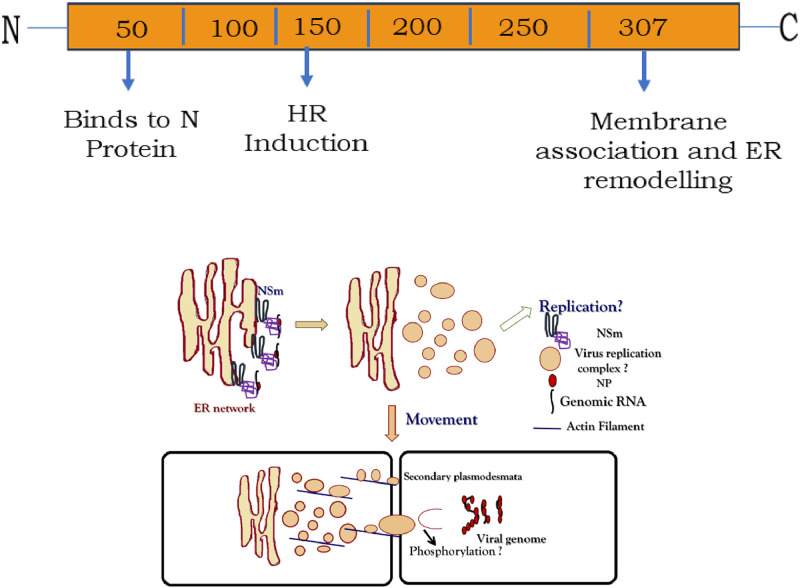
(A) Domain mapping of Tospoviral NSm. The N-terminal unfolded region associates with N protein, The residues100–150 are involved in conferring resistnace and in interaction with SW-5b and C-terminal coiled coil domain associates with ER network and remodels it to vesicles (B) The probable mechanism of cell to cell movement of tospoviruses.

Based on the results available on the cell to cell movement of tospoviruses, the, domains of NSm and a probable mechanism of cell to cell movement in tospoviruses is presented in Fig. 7.

## CRediT authorship contribution statement

**Pratibha Singh:** Writing – review & editing, Writing – original draft, Resources, Funding acquisition. **Rishi Raj:** Writing – review & editing, Visualization. **H.S. Savithri:** Writing – review & editing, Resources, Funding acquisition, Conceptualization.

## Declaration of competing interest

The authors declare the following financial interests/personal relationships which may be considered as potential competing interests: Pratibha Singh reports financial support was provided by Mahatma Gandhi Central University. Pratibha Singh reports financial support was provided by India Ministry of Science & Technology Department of Science and Technology (CRG/2019/2074). H.S. Savithri reports financial support was provided by Indian Institute of Science. H.S. Savithri reports financial support was provided by India Ministry of Science & Technology Department of Biotechnology. H.S. SAvithri reports financial support was provided by India Ministry of Science & Technology Department of Science and Technology. H.S. Savithri reports financial support was provided by Indian National Science Academy. H.S. Savithri reports financial support was provided by The National Academy of Sciences India. Rishi Raj reports financial support was provided by Mahatma Gandhi Central University. Rishi Raj reports financial support was provided by India Ministry of Science & Technology Department of Science and Technology. Pratibha Singh reports a relationship with Mahatma Gandhi Central University that includes: employment. H.S. Savithri reports a relationship with Indian Institute of Science that includes: employment and funding grants. If there are other authors, they declare that they have no known competing financial interests or personal relationships that could have appeared to influence the work reported in this paper.

## Data Availability

No data was used for the research described in the article.
